# Knowledge and attitudes of Chinese medical postgraduates toward research ethics and research ethics committees: a cross-sectional study

**DOI:** 10.1186/s12909-023-04459-y

**Published:** 2023-06-28

**Authors:** Xing Liu, Xianxiong Wang, Ying Wu, Haitao Yu, Min Yang, Kaveh Khoshnood, Esther Luo, Xiaomin Wang

**Affiliations:** 1 Medical Ethics Committee, Xiangya Hospital of Central South University, 87 Xiangya Road, Changsha, 410008 Hunan People’s Republic of China; 2 School of Humanities, Central South University, Changsha, Hunan 410012 People’s Republic of China; 3 Xiangya School of Nursing, Central South University, Changsha, 410013 Hunan People’s Republic of China; 4 Yale School of Public Health, Yale University, 60 College Street, New Haven, CT 06520 USA; 5 Center for Clinical Pharmacology, The Third Xiangya Hospital, Central South University, Changsha, 410013 Hunan People’s Republic of China

**Keywords:** Knowledge, Attitude, Research ethics, Research ethics committees, Medical postgraduates

## Abstract

**Background:**

Research ethics provides the ethical standards for conducting sound and safe research. The field of medical research in China is rapidly growing and facing various ethical challenges. However, in China, little empirical research has been conducted on the knowledge and attitudes of medical postgraduates toward research ethics and RECs. It is critical for medical postgraduates to develop a proper knowledge of research ethics at the beginning of their careers. The purpose of this study was to assess the knowledge and attitudes of medical postgraduates toward research ethics and RECs.

**Methods:**

This cross-sectional study was conducted from May to July 2021 at a medical school and two affiliated hospitals in south-central China. The instrument of the study was an online survey that was distributed via WeChat.

**Results:**

We found that only 46.7% were familiar with the ethical guidelines for research with human subjects. In addition, 63.2% of participants were familiar with the RECs that reviewed their research, and 90.7% perceived RECs as helpful. However, only 36.8% were fully aware of the functions of RECs. In the meantime, 30.7% believed that review by an REC would delay research and make it more difficult for researchers. Furthermore, most participants (94.9%) believed that a course on research ethics should be mandatory for medical postgraduates. Finally, 27.4% of the respondents considered the fabrication of some data or results to be acceptable.

**Conclusion:**

This paper serves to suggest that research ethics education should be prioritized in medical ethics curriculum, and course syllabi or teaching methods should be revised to provide medical postgraduates with a deeper understanding of the principles, regulations, and specifics of research ethics. We also recommend that RECs provide diverse approaches in their review procedure to facilitate the understanding of medical postgraduates of the functions and processes of RECs and to enhance their awareness of research integrity.

## Background

Medical research should adhere to ethical standards that promote and ensure respect for all human subjects while protecting their health and rights [1]. Research Ethics Committees (RECs), also known as Institutional Review Boards (IRBs), are committees of professionals charged with protecting the rights and safety of human subjects in research [2]. RECs are an important element of research ethics, and the primary purpose of RECs is to provide independent review of research proposals to determine whether they meet ethical standards [3]. Given the importance of research ethics, enhancing education in research ethics can help researchers better understand ethical standards and policies as well as improve ethical judgment and decision making to provide safeguards for human subjects [4].

China’s medical research has grown significantly, with regulations in research ethics and RECs being regularly explored. The establishment of RECs in China initially occurred in the field of drug clinical trials. The State Food and Drug Administration (SFDA) issued the Good Clinical Practice (GCP), regulations for drug clinical trials, in 2003 which required research institutions to establish independent RECs [5]. In 2010, the SFDA enacted the Guiding Principles for Ethical Review of Drug Clinical Trials [6] to provide guidance for RECs on developing more operational and standard operating procedures in drug clinical trials. Since then, in 2020, the NMPA in conjunction with the NHC revised the GCP, to establish RECs as separate entities, emphasize and specify the responsibilities of the ethics committee [7]. In addition to the ethical requirements for drug clinical trials, in order to further regulate the ethical review of biomedical research involving human subjects in China, the MOH promulgated the Operational Guideline for Ethic Review of Biomedical Research Involving Human Subjects (For Trial Implementation) in 2007 [8]. The National Health and Family Planning Commission (NHFPC) (now the NHC) issued an updated version in 2016 which described the scope of biomedical research ethics as including research on human subjects and the usage of new medical technologies as well as the collection, usage, or storage of biomedical samples and medical records [9]. In addition, compliance with the code of scientific integrity is the basic requirement for studies to be approved by ethics committees [9]. On the other hand, fabrication or plagiarism in any aspect of research and its results would violate guidelines pertaining to research integrity [10].

Despite continual emphasis by the NMPA and NHC on the development of research ethics regulations and IRBs at the national level, ethical violations still occur, as demonstrated by the Genetically Modified Babies Using CRISPR in China [11]. After this incident, China began stressing the necessity of following ethical principles in research at the legal level [12–15]. However, there remains a lack of attention on research ethics in the field of medical education.

Previous studies outside of China have described the different roles within the medical profession and thus have raised concerns on the importance of their knowledge and attitudes toward research ethics and RECs. For instance, a study in Jordan focused on medical residents’ attitudes towards RECs [16]. Similarly, a Lebanon study focused on physicians as participants [17]. Another study on research ethics in India was conducted amongst dental faculty [18]. However, few studies have focused on medical students or postgraduates. This is an essential group, as it is imperative for these budding clinicians to understand the ethical aspects of research early in their medical education, so that they may conduct safe and responsible research throughout their careers [19]. A study in Myanmar [20] previously showed that fewer post-graduate students were familiar with the function of RECs and principles in research ethics than those who were only aware of an REC. Moreover, 32.8% of participants believed that the fabrication of research data was acceptable and 26.0% thought that REC review would delay research. A study in South India [21] found that undergraduate students had a low level of knowledge towards plagiarism, while a study with Iranian dental students [22] showed that only 44% had good knowledge and 20.8% had a positive attitude towards research ethics. Clearly, there is room for improvement in research ethics education, particularly within the Chinese context.

Little empirical research has been conducted in China on the knowledge and attitudes of medical postgraduates toward research ethics and RECs. With increasing opportunities for physicians to work either in academia or industry, education on research ethics is and will remain vital for those conducting research, and as they are future physicians and scientists, medical students are an important population to target [23]. The ethical knowledge of medical postgraduates is closely related to their education on research ethics, which itself is crucial for implementing sound research. Therefore, it is necessary to assess the knowledge and attitudes of medical postgraduates toward research ethics and RECs in China, to improve outcomes in both medical education and research.

## Methods

### Study design

This cross-sectional study was conducted from May to July 2021 at a medical school and two affiliated hospitals in south-central China and focused on assessing the knowledge and attitudes of medical postgraduates toward research ethics and RECs. In this study, research ethics pertains to research on human subjects, human biological samples, research integrity, etc. WeChat is a popular social media application in China and has thus become an indispensable part of students’ lives in Chinese universities, with a majority of students utilizing the app for communication and socialization [24]. A link to the questionnaire was sent via WeChat to 572 medical postgraduates who had participated in an online medical ethics course during their first or second academic year. This course is mandatory for all medical postgraduates and was launched by an interdisciplinary teaching team with experience in research in clinical medicine, medical ethics, public health, and pharmacy. The purpose of the course, spanning 16 credit hours, is to prepare students in ethics for clinical research. To provide a comprehensive study of medical ethics, course content includes an introduction to medical ethics, theories and applications of medical ethics, ethics of research on medical technology, regulations on medical research ethics, IRB review of medical research, and research integrity.

The link included an informed consent form and questionnaire that could be completed online. After participants read and submitted the initial consent form, they could choose to fill out the questionnaire. The questionnaire was returned anonymously, with no available information to identify the participants with. It took approximately 15 min to complete the questionnaire. We received 572 questionnaires, including 58 from respondents who did not belong to the target population and 514 valid questionnaires.

### The instrument

The questionnaire was derived from a study by Hadir F El-Dessouky et al. [25] and was authorized for translation into Chinese. After translation, four independent professionals with expertise in research ethics assessed the validity of the content and finalized it based on their feedback. The questionnaire consisted of five parts. The first section covered participants’ demographic information, including age, gender, major, education, academic year, ethics training, participation in research projects, and academic publications. The second section asked participants about their knowledge and awareness of research ethics and ethics committees. Questions 1 to 8 could be answered with “Yes”, “No”, “I don’t know” or “I’m not sure” and the other questions were a combination of single-answer and multiple-choice. The third section asked participants about their attitudes toward research ethics, and the fourth section asked about their attitudes toward RECs. For these sections, participants were asked to respond using a Likert scale from 1 to 5 (“strongly agree” = 5, “agree” = 4, “unsure” = 3, “disagree” = 2 and “strongly disagree” =1). In the fifth section, participants were asked for their opinions and recommendations regarding the study of ethics and RECs.

### Sampling method

We used the Cochrane formula (*n* = z2pq/e2) to calculate the sample size with a confidence interval of 0.95 and a margin of error of 5%. The minimum sample size obtained was 385. The total number of questionnaires was increased by 20% to account for the possibility of incorrect completion of the questionnaires or the presence of a large number of missing data. Therefore, the final sample size of the study was estimated to be 462. We selected a total of 514 respondents for our study.

### Data analysis

Analysis was performed using SPSS 26.0 (IBM, Chicago, IL, USA). For descriptive statistics, the categories “strongly disagree” and “disagree” were combined into one category of “disagreement” while “agree” and “strongly agree” were combined into “agreement”. Frequencies and percentages were used to describe categorical variables. The non-parametric Kruskal-Wallis (KW) test was used to assess the correlation between the knowledge and attitudes of medical postgraduates toward research ethics and RECs, with P-values less than or equal to 0.05 being considered statistically significant.

## Results

Of the 572 questionnaires received, 90% were valid after excluding those that did not meet the target population. Table 1 summarizes the demographic information. There were 336 participants from general hospitals and 178 participants from the school of pharmacy. In terms of education, 83.5% were master’s students and 16.5% were doctoral students. Most medical postgraduates (71.4%) reported they had participated in courses or training related to research ethics, and 49.8% (*n* = 256) had led or participated in research projects, while 28.6% (*n* = 147) had not received courses or training related to research ethics. Only 18.3% (*n* = 94) of the participants had published academic papers involving human or animal research.

**Table 1:** Participant demographic characteristics (*n* = 514)

Characteristics	N (%)
**Age (years)**	
18–22	30 (5.8)
23–27	424 (82.5)
28–32	34 (6.6)
33–40	21 (4.1)
> 40	5 (1.0)
**Gender**	
Male	193 (37.5)
Female	321 (62.5)
**Medical school/ Hospital**	
Hospital	336(65.4)
School of Pharmacy	178(34.6)
**Major**	
Internal Medicine	278 (54.0)
Surgery	174 (34.0)
Comprehensive Department	62 (12.0)
**Education**	
Master’s student	429 (83.5)
Doctoral student	85 (16.5)
**Academic year**	
Year 1	444 (86.4)
Year 2	70 (13.6)
**Attended some courses or training related to research ethics**	
Yes	367 (71.4)
No	147 (28.6)
**Hosted or participated in some scientific research projects**	
Yes	256 (49.8)
No	258 (50.2)
**Published some academic papers involving human subject or animal research**	
Yes	94 (18.3)
No	420 (81.7)

**Fig. 1:**
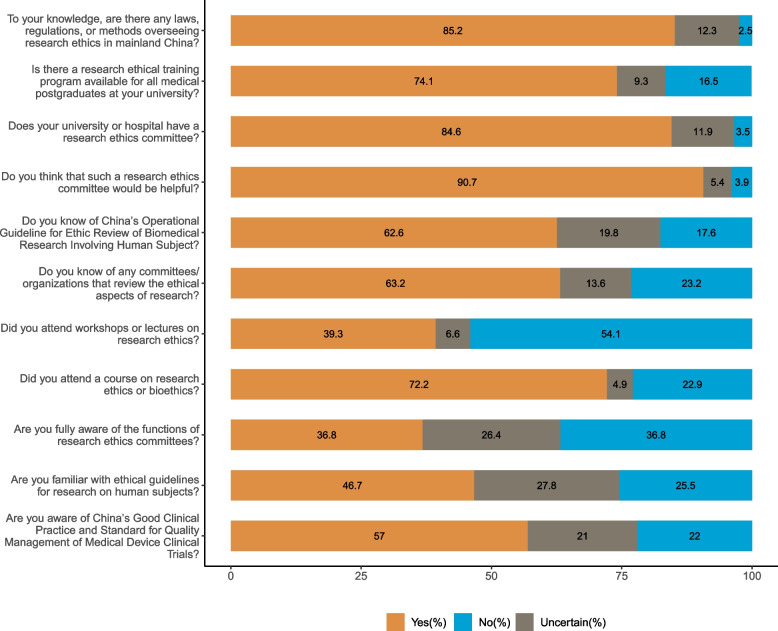
Knowledge and awareness of medical postgraduates regarding research ethics and research ethics committees

As shown in Fig. 1 and 74.1% of the participants reported that their university provided training in research ethics for medical postgraduates and 72.2% had attended courses in research ethics or bioethics. However, only 46.7% were familiar with the ethical guidelines for research with human subjects. We also included a multiple-choice question on research ethics guidelines: If an ethics committee existed in your hospital or university, what did you think was its role? The survey showed that 97.1% of participants thought its role was to review the ethical aspects of research, 90.5% thought it was to safeguard the welfare and rights of research subjects, and 7.8% thought it made research more difficult to conduct.

Figure 1 displays medical postgraduates’ knowledge of RECs and regulations regarding research ethics in China. About 63.2% of participants were familiar with RECs or the departments that reviewed their research, and 90.7% regarded an REC as helpful, but only 36.8% fully understood its function. Although 85.2% knew that there were laws and regulations to regulate research ethics in mainland China, a relatively low proportion understood relevant standards in research ethics. Only 57.0% knew of China’s Good Clinical Practice for and Standard for Quality Management of Medical Device Clinical Trials. Moreover, 62.6% knew about China’s Operational Guideline for Ethic Review of Biomedical Research Involving Human Subject.

Table 2 shows the attitudes of medical postgraduates toward research ethics. Most participants (94.9%) believed that a research ethics course should be mandatory for medical postgraduates, and 96.3% believed that all researchers should receive training in research ethics. In addition, 96.9% thought that measures should be taken to prevent accidental data leakage when obtaining data from research subjects.

Despite existing regulations requiring consent, 35.7% believed that if an investigator wanted to use some of the blood tested in a clinical laboratory for research purposes, informed consent from the patient was not required. Because of concerns that patients might not agree to participate in this study, 30.6% answered that patients should not be informed of potential risks in clinical studies. Furthermore, 27.4% thought that it is sometimes acceptable to fabricate data or results to improve the study, which could be done without harming the patient.

**Table 2:** Attitudes of medical postgraduates toward research ethics

Question	Strongly Agree (%)	Agree (%)	Not Sure (%)	Disagree (%)	Strongly Disagree (%)
Research ethics should be taught as a mandatory module in postgraduate studies.	314 (61.1)	174 (33.8)	17 (3.3)	9 (1.8)	0 (0)
All researchers should be trained in research ethics.	320 (62.3)	175 (34.0)	15 (2.9)	4 (0.8)	0 (0)
There is a need for more emphasis on research ethics in conducting research involving human subjects.	344 (66.9)	155 (30.2)	14 (2.7)	1 (0.2)	0 (0)
When involving our patients in research that presents more than minimal risk, we must seek informed consent from each patient.	330 (64.2)	164 (31.9)	17 (3.3)	3 (0.6)	0 (0)
When obtaining data from research subjects, measures should be taken to prevent accidental disclosure of data.	352 (68.5)	146 (28.4)	10 (1.9)	6 (1.2)	0 (0)
If a blood sample is being obtained for clinical laboratory tests and an investigator would like to use some of this blood for a research study, it is not necessary to obtain informed consent from the patient regarding the research study.	102 (19.8)	81 (15.9)	48 (9.3)	142 (27.6)	141 (27.4)
When conducting clinical research, patients should not be told about potential risks; otherwise, they may not agree to participate in the study.	95 (18.5)	62 (12.1)	13 (2.5)	136 (26.5)	208 (40.4)
It is acceptable sometimes to fabricate some of the data or results to improve the outcome of the research as long as there is no harm to patients.	86 (16.7)	55 (10.7)	16 (3.1)	102 (19.9)	255 (49.6)
It is difficult to get a study published if the researcher does not follow the ethical guidelines.	278 (54.1)	167 (32.5)	21 (4.1)	23 (4.5)	25 (4.8)

Table 3 shows the attitudes of medical postgraduates toward RECs. Most participants (96.1%) agreed that every university or research institution needs an REC to conduct ethical review of research involving humans and laboratory animals; 97.3% believed that research involving humans must be reviewed by an REC, and 98.0% believed that human research should first be reviewed by an REC before a scientific committee. However, 23.9% thought that ethical review was only for international, collaborative research and projects, and 24.9% believed that review of research by both a scientific committee and an REC was not necessary. In addition, 30.7% answered that REC review would delay research and make it more difficult for researchers.

**Table 3:** Attitudes of medical postgraduates toward research ethics committees

Question	Strongly Agree (%)	Agree (%)	Not Sure (%)	Disagree (%)	Strongly Disagree (%)
Every university or research institution needs a research ethics committee to conduct an ethical review of research involving humans and laboratory animals.	337 (65.6)	157 (30.5)	16 (3.1)	4 (0.8)	0 (0)
Research involving humans must be reviewed by a research ethics committee.	360 (70.0)	140 (27.3)	12 (2.3)	2 (0.4)	0 (0)
Human research must first be reviewed by a research ethics committee before it can be reviewed by a scientific committee.	348 (67.7)	156 (30.3)	8 (1.6)	1 (0.2)	1 (0.2)
Ethical review enhances the credibility of research.	322 (62.6)	149 (29.0)	36 (7.0)	7 (1.4)	0 (0)
Ethical review is only for international collaborative research and projects.	73 (14.2)	50 (9.7)	33 (6.4)	183 (35.6)	175 (34.1)
Because there are scientific committees, research ethics committees are not necessary to review research.	74 (14.4)	54 (10.5)	13 (2.5)	175 (34.1)	198 (38.5)
The reviews of research ethics committees will delay research and make it more difficult for researchers.	73 (14.2)	85 (16.5)	71 (13.8)	149 (29.0)	136 (26.5)
Research ethics committee members should be trained in research ethics.	335 (65.2)	145 (28.2)	16 (3.1)	11 (2.1)	7 (1.4)
The members of the research ethics committee should at least be professors with high authority in universities.	260 (50.6)	163 (31.7)	60 (11.7)	24 (4.7)	7 (1.3)
In order to gain confidence in research ethics committee decisions, research ethics committees must be subject to oversight from some higher authorities.	322 (62.6)	164 (31.9)	17 (3.3)	4 (0.8)	7 (1.4)

The correlation between knowledge and attitudes of medical postgraduates towards research ethics and RECs is shown in Table 4. Participants who were familiar with ethical guidelines for research with human subjects had higher scores than those who were not familiar (*p* < 0.05). In addition, participants who attended workshops or lectures on research ethics had a stronger attitude toward research ethics and RECs. These participants thought that RECs would be helpful and had higher scores than those who did not attend workshops or lectures on ethics (*p* < 0.05). Similarly, those who knew of *China’s Operational Guideline for Ethic Review of Biomedical Research Involving Human Subject* toward research ethics had a stronger attitude than those who were not familiar with the guideline (*p* < 0.05).

**Table 4:** Correlation between knowledge and attitudes of medical postgraduates toward research ethics and research ethics committees

Question	Research ethics		Research ethics committees	
	Median (IQR)	*p*-value*	Median (IQR)	*p*-value*
**Are you familiar with ethical guidelines for research on human subjects?**				
Yes	3.67 (3.59-4.00)	0.005	3.90 (3.70–4.20)	0.003
No	3.67 (3.33-4.00)		3.80 (3.50-4.00)	
Uncertain	3.78 (3.44-4.00)		3.80 (3.60-4.00)	
No vs. Uncertain		0.115		1.000
No vs. Yes		0.003		0.013
Uncertain vs. Yes		0.973		0.018
**Did you attend a course on research ethics or bioethics?**				
Yes	3.67 (3.44-4.00)	0.076	3.80 (3.60–4.10)	0.154
No	3.67 (3.33-4.00)		3.80 (3.60-4.00)	
Uncertain	4.00 (3.50–4.56)		3.80 (3.40-4.00)	
**Did you attend workshops or lectures on research ethics?**				
Yes	3.78 (3.67–4.28)	0.000	3.90 (3.60–4.20)	0.014
No	3.67 (3.33-4.00)		3.80 (3.60-4.00)	
Uncertain	3.78 (3.33–4.11)		3.80 (3.58-4.00)	
No vs. Uncertain		0.827		1.000
No vs. Yes		0.000		0.022
Uncertain vs. Yes		1.000		0.176
**Is there a research ethical training program available for all medical postgraduates at your university?**				
Yes	3.67 (3.44-4.00)	0.809	3.80 (3.60–4.10)	0.985
No	3.67 (3.33-4.00)		3.90 (3.45–4.20)	
Uncertain	3.67 (3.33-4.00)		3.80 (3.56–4.18)	
**Do you know of any committees/ organizations that review the ethical aspects of research?**				
Yes	3.67 (3.56-4.00)	0.230	3.80 (3.60–4.10)	0.171
No	3.67 (3.33-4.00)		3.80 (3.40-4.00)	
Uncertain	3.67 (3.33–4.14)		3.80 (3.50–4.10)	
**Do you think that such a research ethics committee would be helpful?**				
Yes	3.67 (3.44-4.00)	0.099	3.80 (3.60–4.10)	0.003
No	3.44 (3.22-4.00)		3.65 (3.13-4.00)	
Uncertain	3.62 (3.22-4.00)		3.70 (3.33–3.98)	
No vs. Uncertain				1.000
No vs. Yes				0.044
Uncertain vs. Yes				0.040
**Are you fully aware of the functions of research ethics committees?**				
Yes	3.67 (3.67–4.22)	0.001	3.80 (3.70–4.20)	0.019
No	3.67 (3.33-4.00)		3.80 (3.60-4.00)	
Uncertain	3.67 (3.33-4.00)		3.80 (3.50–4.10)	
No vs. Uncertain		1.000		1.000
No vs. Yes		0.002		0.025
Uncertain vs. Yes		0.010		0.109
**Does your university or hospital have a research ethics committee?**				
Yes	3.67 (3.44-4.00)	0.415	3.80 (3.60–4.10)	0.063
No	3.73 (3.19-4.00)		3.80 (3.20-4.00)	
Uncertain	3.67 (3.33–4.06)		3.80 (3.40-4.00)	
**To your knowledge, are there any laws, regulations, or methods overseeing research ethics in mainland China?**				
Yes	3.67 (3.44-4.00)	0.328	3.80 (3.60–4.10)	0.310
No	3.56 (3.28–4.34)		3.80 (3.35–4.20)	
Uncertain	3.67 (3.33-4.00)		3.80 (3.50-4.00)	
**Are you aware of China’s Good Clinical Practice and Standard for Quality Management of Medical Device Clinical Trials?**				
Yes	3.78 (3.56–4.11)	0.001	3.80 (3.70–4.10)	0.001
No	3.67 (3.33-4.00)		3.90 (3.60–4.10)	
Uncertain	3.67 (3.33–3.89)		3.80 (3.40-4.00)	
No vs. Uncertain		0.514		0.018
No vs. Yes		0.000		0.000
Uncertain vs. Yes		0.105		1.000
**Do you know of China’s Operational Guideline for Ethic Review of Biomedical Research Involving Human Subject?**				
Yes	3.78 (3.56–4.11)	0.000	3.80 (3.60–4.10)	0.039
No	3.56 (3.33-4.00)		3.80 (3.60–4.03)	
Uncertain	3.67 (3.33-4.00)		3.80 (3.50-4.00)	
No vs. Uncertain		1.000		0.865
No vs. Yes		0.000		0.829
Uncertain vs. Yes		0.002		0.038

*IQR* Interquartile range

*Kruskal-Wallis test was used

## Discussion

Research on human subjects has been increasing in China, both in magnitude and significance. In 2021, the Center for Drug Evaluation received 11,658 registration applications, an increase of 13.79% from the previous year [26]. Research continues to require appropriate ethical oversight by RECs, a requirement that China has duly acknowledged in its national laws and regulations. However, this is the first study in China to use this particular questionnaire to assess the knowledge and attitudes of medical postgraduates toward research ethics and RECs. According to our findings, participants showed high levels of knowledge and positive attitudes toward ethics in general.

About 71.4% (*n* = 367) of participants had participated in courses or training related to research ethics. But only 46.7% were familiar with the guiding principles of research ethics with human subjects. Therefore, medical schools should increase their efforts to develop and disseminate courses and training related to research ethics and make such training mandatory and available to all medical postgraduates [27]. Revising course syllabi or adjusting teaching methods might help medical postgraduates develop a deeper understanding of the principles, regulations, and specifics of research ethics. For instance, a study in Jordan recommended that RECs should conduct educational seminars to discuss the function of RECs and their review processes [2].

### Knowledge of research ethics and research ethics committees

In our study, medical postgraduates displayed a high rate of correct answers (greater than 80%) to questions regarding informed consent from patients, vulnerable groups, as well as in the use of biological samples in research ethics. This may be attributed to the enhanced regulation of clinical research ethics in China, as well as ethical review and informed consent norms being legalized after 2019, including for biological samples. These regulations note that the collection of human genetic resources in China should be subject to the prior written informed consent of a provider, with the specification that human genetic resources include human genetic resource materials and information [28].

Although 84.6% of the participants in our study claimed that their university or hospital had a research ethics committee, only 36.8% were fully aware of the function of the ethics committee. Similarly, less than one-third of participants surveyed in a study involving Middle Eastern dental schools said they were familiar with the function of an REC [25]. A study in Lebanon showed that nearly 40% of its participants were not acquainted with of the function of an REC [17]. Lastly, only 47.1% of postgraduate medical students in Myanmar were fully aware of the function of an REC [20]. Protecting research participants is an integral function of IRBs [29]. Moreover, RECs or IRBs are responsible for the independent evaluation of proposed research and ultimately for ensuring that research meets ethical standards and regulations [30]. Medical postgraduates should thus increase their familiarity with IRB functions to produce strong and ethical research.

The present study showed that only 61.7% of medical postgraduates correctly answered the question about international medical research ethics guidelines. In comparison, a study in Myanmar noted that less than half of the graduate students knew that common guidelines existed in research ethics (36.3%) [20]. In our study, only 57.0% of the medical postgraduates were conscious of China’s Good Clinical Practice and Standard for Quality Management of Medical Device Clinical Trials, and only 62.6% of the students knew of China’s Operational Guideline for Ethic Review of Biomedical Research Involving Human Subjects. These results indicate that some medical students are not familiar with local Chinese laws and regulations on research ethics. Medical schools should raise the level of students’ knowledge regarding local and international ethical regulations, so that they can conduct research responsibly.

### Attitudes toward research ethics

Although there was no significant difference between scores (Median (IQR)) on attitudes toward research ethics and having published academic papers involving human or animal research, those who had published academic papers scored higher. This indicates that students with research experience possessed a somewhat deeper understanding of research ethics, which might have contributed to their capabilities to conduct ethical research.

About 35.7% of the participants agreed that if some blood was to be used for research purposes in a clinical laboratory, then obtaining a patient’s informed consent was unnecessary. Similar results were found in Myanmar, in which 20% of their respondents agreed that performing research on blood samples collected for clinical purposes without obtaining specific consent was appropriate [20]. However, there are provisions in the Chinese code of ethics regarding specific situations for consent and exemptions in consent. Specifically, only two scenarios allow for the exemption of consent, according to the Operational Guideline for Ethic Review of Biomedical Research Involving Human Subject [9]. To strengthen the management of human biological samples for scientific research in medical and health institutions, the NHC stipulated that the acquisition of biological samples should not be exempted from ethical review and must be carried out after obtaining the informed consent of the donor or a guardian [31]. Institutions should develop effective strategies to standardize the informed consent process and provide formal training for and supervision in obtaining informed consent [32]. Hence, the specific use of informed consent requires more detailed guidance and training for medical students, to deepen their understanding of the purpose of informed consent and minimize the gap between students’ awareness and comprehension, with the promotion of regulations in informed consent.

Cases of research misconduct, transgressions related to research ethics and research integrity, and forms of ethically questionable behaviors have been frequently published [33]. Moreover, occurrences of misconduct in research such as falsification, plagiarism, inappropriate authorship, and duplicate submissions have been reported in China, emphasizing the need to address these issues [34]. Misconduct within China also includes problems with publications, grant applications, and other ethical issues [35]. Overall, China has been facing serious challenges with scientific integrity. Although the country has published 8% of the world’s scientific articles, by 2017, it had garnered 24% of all retractions [36]. In order to further strengthen the construction of research integrity in the field of medical research, in 2021, the NHC, MOST, and National Administration of Traditional Chinese Medicine jointly revised the Medical Research Integrity and Related Codes of Conduct, which highlighted the main responsibility of scientific research integrity in medical research institutions [37]. Twenty-two departments, including the MOST, issued the Rules for the Investigation and Handling of Untrustworthy Behaviors in Scientific Research, which further standardized the investigation procedures, unified the handling standards, and contained more operational norms for the investigation and handling of untrustworthy conducts in scientific research [38]. Therefore, universities must build on these regulations and examine how to provide effective education in scientific integrity to students conducting research [36].

Regarding research misconduct, 27.4% of the respondents believed that it was acceptable to fabricate some data to improve results, which could be done without harm to patients. Similarly, a Myanmar study showed that 32.8% agreed that it was acceptable to manufacture research data to improve results [20]. Although this rate from our study is lower than that of other countries, it demonstrates how research misconduct among medical students still often occurs. A recent survey found that Ph.D. students had the most difficulty in meeting the standards of responsible research. 53% of them admitted to frequently engaging in one of the 11 questionable behaviors in research within the past three years [39]. Thus, research misconduct by medical postgraduates deserves further study and vigilance. Abiding by research ethics is the cornerstone of scientific research, and a lack of research integrity would only hinder China’s growth in conducting original science, damage the reputation of Chinese academics, and dampen the impact of scientific knowledge developed in China [40]. It is crucial to strengthen training in scientific research integrity, continue to build a research governance system and improve the evaluation system for research.

### Attitudes toward research ethics committees

The responsibilities of RECs include the scientific and ethical nature of reviewing research [41]. Establishing a human subject protection system based within RECs with collaborative review between departments will be of great benefit to the quality and efficiency of REC operations including project reviews [42]. However, 30.7% of participants in our study believed that REC review would delay research and make work more difficult for researchers. These findings were similar to those from the Myanmar [20] and Iranian studies [22]. To clear such misconceptions, RECs should function in a transparent and accountable manner, while promoting their training programs so that medical students will clearly understand that IRBs do not hinder research.

In our study, participants’ attitudes towards research ethics and RECs were significantly associated with their knowledge of ethics principles. These findings may demonstrate how medical postgraduates who are more familiar with or knowledgeable in research ethics and its principles, as well as RECs, may also display stronger attitudes towards these areas. Thus, educating these students in ethics principles early in their research careers will help them to achieve a proper balance between the practice and principles of research ethics and equip them to resolve ethical dilemmas in their future clinical research.

### Limitations

We acknowledge that this study may have some limitations. There is no significant difference in REC attitude scores (*P* > 0.05) for the different characteristics of the participants (such as age, gender, etc.) included in the study; further research should consider the stratification of demographic data to reduce homogeneity. Moreover, questions may not cover all ethical problems within human subjects research but instead represent the wide range of basic information in research ethics.

## Conclusion

This study was designed to assess medical postgraduates’ knowledge of and attitudes toward research ethics and RECs, as they represent future physician scientists. It is the duty of the physician to promote health and safeguard the rights of patients when the physician is involved in medical research. The knowledge and conscience of the physician should be committed to this duty [43].

This study highlighted three areas that we should further improve: First, medical universities should strengthen the construction of research ethics courses and modify syllabi or teaching methods for medical postgraduates, so that students can have a deeper understanding of Chinese laws and international regulations. Second, RECs should provide diverse ways within their review procedure to facilitate medical postgraduates’ comprehension of the functions and process of RECs. Lastly, the MOST needs to cultivate an atmosphere of scientific research integrity to raise medical postgraduate students’ awareness and willingness to ensure sound and ethical research.

## Data Availability

The datasets used and/or analyzed during the current study are available from the corresponding author on reasonable request.

## Abbreviations

IRB: Institutional Review Board
REC: Research Ethics Committee
HRPP: Human Research Protection Programs
MOH: Ministry of Health
NHFPC: National Health and Family Planning Commission
SFDA: State Food and Drug Administration
NMPA: National Medical Products Administration
GCP: Good Clinical Practice
NHC: National Health Commission
ICH: The International Council for Harmonization of Technical Requirements for Pharmaceuticals for Human Use
CDE: Center for Drug Evaluation
NPC: National People’s Congress
CIOMS: Council for International Organizations of Medical Sciences
WMA: World Medical Association
SPSS: Statistical Product and Service Solutions
MOST: Ministry of Science and Technology.
